# *Hermetia illucens* Larvae Reared on Different Substrates in Broiler Quail Diets: Effect on Physicochemical and Sensory Quality of the Quail Meat

**DOI:** 10.3390/ani9080525

**Published:** 2019-08-02

**Authors:** Marco Cullere, Michael Josias Woods, Liesel van Emmenes, Elsje Pieterse, Louwrens Christiaan Hoffman, Antonella Dalle Zotte

**Affiliations:** 1 Department of Animal Medicine, Production and Health, University of Padova, Agripolis, Viale dell’Università 16, Legnaro, 35020 Padova, Italy; 2 Department of Animal Sciences, Stellenbosch University, Matieland 7602, South Africa; 3 Centre for Nutrition and Food Sciences, Queensland Alliance for Agriculture and Food Innovation (QAAFI), University of Queensland, Health and Food Sciences Precinct, 39 Kessels Rd, Coopers Plains 4108, Australia

**Keywords:** black soldier fly larvae, substrate, quail, insect meal, alternative protein source, animal feeding, meat quality, proximate composition, fatty acids, amino acids

## Abstract

**Simple Summary:**

*Hermetia illucens* (HI) is one of the most promising insect species that can be exploited as alternative and sustainable feed ingredients in poultry farming. HI larvae can grow on a wide range of decomposing organic substrates, thus transforming organic waste into high-value feed products. When HI is included in the diets of different poultry species, it provides positive results in terms of productive performance, the health status of the animals, and overall product quality. However, a common drawback highlighted by most studies has been a suboptimal quality of the meat lipids of HI-fed poultry (high amount of saturated fatty acids (SFAs) to the detriment of polyunsaturated fatty acids (PUFA)). The present experiment tested the effects of a dietary inclusion of HI larvae farmed on two different substrates in the diets of meat quails. The two substrates were 100% layer mash (conventional rearing substrate) and 50:50 layer mash/fish offal, which aimed to increase the PUFA content of the larvae. The overall results showed that it was possible to improve the fatty acid profile of the larvae by modulating the rearing substrate, which in turn improved the lipid quality of the meat obtained from quails fed diets containing such larvae.

**Abstract:**

This research aimed at improving the fatty acid (FA) profile of *Hermetia illucens* larvae (HI) and evaluating the effects of their inclusion in growing broiler quails’ diets on the meat physicochemical quality, including detailed amino acid (AA) and FA profiles, sensory traits, and retail display. HI larvae were reared on two different substrates: layer mash (HI1) and 50:50 layer mash/fish offal (HI2). A total of 300 10-day-old quails were allocated to the three dietary groups (five replicates/each): a soybean meal-based diet was formulated (Control), and two other diets were formulated that included either 10% HI1 or HI2. Quails were fed the experimental diets until slaughter. Diets were formulated to be isonitrogenous and isoenergetic. Breast meat quality was affected by the dietary treatments, which displayed different proximate compositions and AA and FA profiles. Meat physical quality, sensory profile, and retail display remained unaffected for the most part. Overall, results showed that it is possible to improve the FA profile of the HI-fed quails’ meat and thus lipid quality through substrate modulation of the HI’s diet.

## 1. Introduction

Quails are birds of economic importance due to a series of positive features such as high meat quality and egg production, as well as fast returns on investment, which is possible due to their early sexual maturity, rapid growth, short generation interval, high laying rate, and limited feed and space required/bird [1]. As for other poultry species, commercial feed formulations mainly rely on soybeans as protein and fat sources, which require a vast amount of water and land to grow and thus have a significant environmental impact [2]. Due to growing demographic trends and the consequent augmented pressure on natural resources such as water and land (deforestation), as well as an ever-increasing feed–food competition, the search for novel feed ingredients for poultry diets with the potential to improve the sustainability of the sector is of utmost importance. From this perspective, insects have been recognized as one of the possible candidates to solve this issue. This was internationally emphasized for the first time with the first International Conference on insects for food and feed, organized by the Food and Agriculture Organization of the United Nations in 2014. In the following years, research studies on this topic as well as innovations and applications of techniques by the industry have grown tremendously [3]. In addition, the European legislative framework regarding the use of insects in animal nutrition was updated, which led to the publication of Regulation No. 2017/893, which allows the use of seven insect species in aquaculture. Despite this key success, more research efforts are required to allow for their utilization in poultry feeding, where insect consumption already falls within their natural dietary habits, particularly in free-range production systems [4].

One of the most promising insect species in this sense is the black soldier fly (*Hermetia illucens*, HI), a Diptera of the Stratyomidae family. Together with their undoubtfully interesting nutritional profile [5] and suitability for mass production, *H. illucens* larvae can exploit a wide range of decomposing organic material for their growth, thus representing an opportunity to recycle organic matter into valuable nutrients [6]. Independent of the inclusion level, when tested in the diets of different animal species (including fish [7], poultry [8,9,10,11], pig [12], and rabbit [13,14] species), overall results such as the health status of the animals, productive performances, and the overall quality of the derived animal products (i.e., meat and eggs) proved to be satisfactory. However, a constant nutritional drawback emerged related to the fatty acid (FA) profile of the animal product derived from animals fed with larvae of this Dipteran. In fact, *H. illucens* larvae are naturally rich in saturated fatty acids (SFAs; mainly lauric, myristic, and palmitic) and poor in unsaturated FA [15]. For this reason, they are suboptimal in providing healthy food for modern consumers. As previous research has demonstrated that the rearing substrate can have a great impact on the chemical composition of the larvae [16], the hypothesis is that the FA profile of *H. illucens* larvae could be improved by rearing them in a substrate enriched with a source of *n*-3 polyunsaturated FA (PUFA), recycling a relevant nutrient source that is considered a byproduct: fish offal. The ultimate goal is the successful application of such larvae to poultry nutrition, aiming to obtain a meat with a healthy FA profile. The only research on this topic published to date found promising results [17]. However, the study concerned a possible application of *n*-3-enriched larvae in fish diets, whereas no scientific works are available on poultry species. Therefore, the present research project studied the inclusion of 10% HI larvae reared on a conventional substrate or on a substrate enriched with *n*-3 PUFA in the feed of broiler quails, aiming to improve the FA profile of quails’ meat. In the first report of this study [18] the total tract nutrients’ digestibility and feed-choice as well as the quails’ productive performance and carcass traits were studied: despite feed-choice and carcass weight were not in favor of the group including the *n*-3 enriched larvae, nutrients’ digestibility, mortality and carcass yield were satisfactory. In this, the second part of the study, detailed meat quality aspects, including physical characteristics, proximate composition, cholesterol, heme iron and amino acid contents, the FA profile, sensory traits, and the storage stability of the quails fed these diets, were evaluated.

## 2. Materials and Methods 

The present trial was conducted after approval by the veterinary authority and according to Article 2, DL 4 March 2014, No. 26, of the Official Journal of the Italian Republic (http://www.gazzettaufficiale.it/eli/id/2014/03/14/14G00036/sg), which implements the EU Directive 2010/63/EU on the protection of animals used for scientific purposes. 

### 2.1. Experimental Design

For the present experiment, three dietary treatments were tested on broiler quails from 10 to 29 days of age: a control diet (Control (C)) formulated according to the conventional grower diet used on the farm and two diets containing 10% dried and ground black soldier fly (*Hermetia illucens*) larvae as a partial substitution for conventional protein/fat sources. The larvae included in the two diets differed, as they were farmed on two different growth media: the first was a conventional substrate made of 100% chicken layer mash (HI1), whereas the second was made of 50% chicken layer mash and 50% fish offal (HI2), which was aimed at increasing larvae *n*-3 PUFA content. Detailed information about insect farming, diet formulation and chemical composition, quail farming conditions, live performances, and slaughtering procedure are provided in Woods et al. [18]. Each experimental group consisted of 100 quails (20 birds/cage, 5 cages/dietary treatment). All slaughtered quails (*n* = 99, *n* = 97, and *n* = 97 from the C, HI1, and HI2 groups, respectively) were considered for meat quality evaluations. From each slaughtered bird, breasts were excised and analyzed at the Laboratory of the Department of Animal Medicine, Production and Health (MAPS) of the University of Padova (Italy). Each breast was labeled, vacuum-packaged in polyethylene bags (water vapor transmission rate: 3.5 ± 1 g/m^2^ day at 23 °C and 85% ± 2% relative humidity) using a CSV-41n ORVED (Orved S.p.A., Musile di Piave, Venice, Italy) machine (99% vacuum level), and stored at −40 °C until analytical determinations. For every experimental dietary group, 24 breasts were dedicated to an analysis of the proximate composition, cholesterol content, and amino acid and fatty acid (FA) profiles; 10 breasts were for heme iron content; 16 breasts were for the sensory profile; and 12 breasts were for a retail display trial. The remaining breasts were dedicated to physical evaluations. 

### 2.2. Physical Meat Quality

After 2 weeks of frozen storage, quail breasts were weighed, allowed to thaw for 12 h at 4 °C, and weighed again to calculate the thawing loss (%). Color measurements were performed on the cranial and caudal part of the *Pectoralis major* muscle using a RM200QC colorimeter (X-Rite Co, Neu-Isenburg, Germany; measuring area, 8 mm; measuring geometrics, 45/0 image capture; Illuminant/Observer D65/10) and considered lightness (L*), redness (a*), and yellowness (b*) indexes [19]. In the same parts of the breast considered for color determination, pH was measured with a portable pH meter (FG2-Five GoTM; Mettler Toledo, Greifensee, Switzerland) calibrated at pH 4.0 and 7.0. Color and pH values represented the average of two repeated measurements. Breasts were then vacuum-sealed using the above-mentioned equipment and cooked in a water bath set at 80 °C until the core temperature reached 77 °C. Then, samples were cooled in an iced bath, gently dried by dabbing with a paper towel, and weighed to compute the cooking and the total losses (%). Shear force was assessed with a TA-HDi Texture Analyzer (Stable Macro System, London, UK) on four cooked meat cores (diameter 1.25 cm) per sample and sheared perpendicularly to the muscle fiber direction with a Warner–Bratzler cell (100 kg load cell, 2 mm/s crosshead speed) fitted on the texturometer. Warner–Bratzler shear force (WBSF) was calculated by averaging four measurements/sample.

### 2.3. Chemical Meat Quality

The heme iron content of fresh breast meat samples was determined following the method described by Hornsey [20] and was expressed as mg He-Fe/kg of fresh tissue. Breasts dedicated to the other chemical determinations were randomly paired (to guarantee enough material to perform the scheduled analyses) and ground with a Retsch (Retsch Italia Verder Scientific S.r.l. Torre Boldone, BG, Italy) Grindomix GM 200 (7000 g for 10 s). In this way, 12 samples/experimental group were obtained. Once ground, meat samples were frozen at −40 °C, freeze-dried, and ground again (7000 g for 5 s) to obtain a fine powder, which was used to determine proximate composition, amino acid and FA profiles, and cholesterol content. The proximate composition of breast meat samples was analyzed in accordance with the Association of Official Analytical Chemists (AOAC) [21] methods. The cholesterol content was determined through an absolute quantitative analysis using HPLC, following the method described by Casiraghi et al. [22]. The amino acid content of the experimental diets as well as that of the meat samples was analyzed according to the methods described by Cunico [23]. During this process, a sample weighing 0.1 g was placed in a specialized hydrolysis tube. Six mmilliliters of hydrochloric acid (HCl) solution and 15% phenol solution was then added to the sample. The tubes were then vacuated, and nitrogen (N) was added under pressure. The tubes were subsequently sealed off with a blue flame, and the samples were left to hydrolyze at 110 °C for 24 h. After hydrolysis, the samples were transferred to Eppendorf tubes and refrigerated until being sent to the Central Analytical Facility of Stellenbosch University. Amino acid composition was determined by means of the Waters AccQ Tag Ultra Derivatization method. The quantitative results are expressed as g/100 g meat.

### 2.4. Fatty Acid Profile of Hermetia illucens Larvae, Diets, and Meat Samples

The lipid extraction was performed by accelerated solvent extraction (M-ASE) using different solvents according to the following specific matrix: petroleum ether for HI larvae and diets and chloroform/methanol 1:2 for breast meat samples. Total lipid content was determined gravimetrically after the removal of the solvent by evaporation under nitrogen stream at 50 °C. Samples were subsequently transmethylated using a methanolic solution of H_2_SO_4_ (4%) in order to determine fatty acid methyl esters (FAMEs). A biphasic separation was obtained by adding 0.5 mL of distilled water and 1.5 mL of *n*-Heptane to each sample. FAMEs were quantified by a gas chromatographer (Shimadzu GC17A) equipped with an Omegawax 250 column (30 m × 0.25 μm × 0.25 μm) and a Flame Ionization Detector. Helium was used as the carrier gas at a constant flow of 0.8 mL/min. The injector and detector temperatures were 260 °C. Peaks were identified based on commercially available FAME mixtures (37-Component FAME Mix, Supelco Inc., Bellefonte, PA, USA). The results were expressed as % of total detected FAME. The FA compositions of the *H. illucens* larvae and diets are presented in Table 1.

### 2.5. Sensory Analysis

Meat was analyzed at the Sensory Analysis Division of the “Istituto per la Qualità e le Tecnologie Agroalimentari, Laboratorio Analisi Sensoriale (Veneto Agricoltura)”, Thiene, Vicenza, Italy. Quail breasts were subjected to a descriptive sensory analysis to detect possible differences between the dietary treatments (C vs. HI1 vs. HI2). The sensory analysis was performed by an eight-member trained panel who were qualified as experts according to ISO 8586 and had experience with descriptive tests (ISO 13299) on various food matrixes. All judges who perform tests with accredited methods undergo training every 3 years. Panelists underwent two pretest training sessions of 1 h each to familiarize themselves with the matrix and select appropriate descriptors and possible off-odors and off-flavors, which were also drawn from the literature. The meat used for the training sessions (quail breasts) was purchased at a local supermarket, and it was processed, stored, handled, and cooked in the same manner as the samples that were used for the subsequent sensory analysis. The panel received a list of descriptors to score on numerical and continuous scales from 0 (the lowest score for each attribute) to 10 (the highest score for each attribute). Olfactory, gustative, and textural aspects were evaluated. The descriptors were odor intensity, off-odor intensity, flavor intensity, off-flavor intensity, juiciness, toughness, chewiness, and fibrousness. The chosen off-odors and off-flavors were game meat, metallic, liver, oil/fat, peanut/hazelnut, and rancid. For the experiment, a total of 16 quail breasts/treatment were used, and 2 days of analysis were scheduled (8 quail breasts/treatment per session). After 1 month of frozen storage at −40 °C, quail breasts were allowed to thaw for 16 h at +4 °C. Each sample was then placed on a cooking plate (model GR6010 XL Health Comfort, 2400 Watt; Rowenta, Erbach, Germany) set at thermostat position “2” and cooked for 5 min/side until the core temperature reached 74 °C. Subsequently, samples were individually wrapped in aluminum foil, placed in aluminum trays, and served to the panel in random sequence. Samples were identified by a random three-digit code. The evaluation sheet, distribution of samples to the judges, and data acquisition were performed using FIZZ software (Biosystèmes France, St. Ouen l’Aumône, France) installed in eight terminals in the tasting booths of the laboratory. In addition, for each sample, panelists were asked to indicate if and which of the listed off-odors and off-flavors they could recognize. Still water at room temperature and unsalted crackers were available to panelists for palette cleansing throughout each sensory session.

### 2.6. Retail Display Trial

Quail breasts were individually wrapped with food-grade polyvinyl chloride (PVC) film (thickness: 8.5 µm ± 8%; breaking load: >17 N/mm; extension: >135%; temperature range: from −30 °C to +40 °C) to prevent direct air contact and stored in a refrigerator for an eight-day retail display trial. The refrigerator was set at 4 °C ± 1 °C, and continuous cold fluorescent light illumination (L58 W⁄840 LUMILUX Cool White, Osram, Germany) was provided for the whole period to simulate commercial retail conditions. Analyses were performed at days 0 (24 h postmortem) and 8 of refrigerated storage. For storage drip loss calculation, the quail breasts were removed from the refrigerator, unwrapped, and weighed. The pH and L*a*b* color values were measured (in duplicate) on the same breast portions using the same instruments described previously. After physical evaluation, the breasts were ground with a Retsch Grindomix GM 200 (7000 g for 10 s) and were used to determine the extent of muscle lipid oxidation. The latter was evaluated with a spectrophotometer (Hitachi U-2000; Hitachi, Mannheim, Germany) set at 532 nm that measured the absorbance of thiobarbituric acid-reactive substances (TBARs) and a 1,1,3,3-tetraethoxypropane calibration curve [24]. Oxidation products were quantified as malondialdehyde (MDA) equivalents (mg MDA/kg muscle).

### 2.7. Statistical Analysis

Meat physical traits, proximate composition, heme iron, cholesterol, amino acid content, and FA profile data were subjected to a one-way ANOVA with the experimental diet (C, HI1, and HI2) as a fixed effect, following the GLM procedure of the SAS 9.1.3 Statistical Analysis Software (Cary, NC, USA) for Windows [25]. A mixed model (PROC MIXED) was used to detect any dietary influence on sensory analysis scores, considering experimental diet and the eight panelists as fixed and random effects, respectively. Least square means were obtained using the Bonferroni test, and the significance was calculated at a 5% confidence level. A χ^2^ test using the Marascuilo [26] procedure was performed on off-odor and off-flavor characterization to detect the differences between treatments.

## 3. Results

Overall, quail breasts derived from birds fed with diets containing 0% or 10% dried *H. illucens* larvae reared on two different growing media (HI1 and HI2) showed similar physical meat quality traits to those of the quails fed the control diet (Table 2). Specifically, breasts displayed similar pH values (*p* > 0.05), Commission Internationale de l’Éclairage L*a*b* color values (*p* > 0.05), and thawing losses (*p* > 0.05). The only exception was the cooking loss percentage, which was highest in quail breasts of the HI2 group (*p* < 0.05); however, this did not affect the total loss percentage (*p* > 0.05) nor the meat toughness (*p* > 0.05), which was similar in the three experimental groups.

The chemical composition of quail breasts was significantly affected by the tested experimental diets (Table 3). Breasts of the HI1 group displayed the highest lipid content (5.27% vs. 4.89% and 4.93% for HI1, C, and HI2 breast meat, respectively; *p* < 0.01) to the detriment of the protein fraction (*p* < 0.05), which was lower than that of C but not different from HI2. Independent of the growing substrate used to farm *H. illucens* larvae, their inclusion in the quails’ diets increased the cholesterol content of the breast meat compared to the C group (*p* < 0.01). Water, ash, and heme iron contents were not affected by the dietary treatment.

The protein percentage as well as the majority of the essential and nonessential amino acids (g/100 g meat) of quail breast meat was affected significantly by the dietary treatments (Table 4). The 10% dietary inclusion of HI1 lowered the protein percentage (on a dry mass, defatted basis) of quails’ breast meat compared to C, whereas HI2 meat showed an intermediate result (81.4%, 83.4%, and 89.5% for HI1, HI2, and C breast meat, respectively; *p* < 0.05). The above-mentioned result was mainly attributable to the low amounts of the essential amino acids histidine, leucine, methionine, and phenylalanine. The rearing substrate of the larvae played a role in determining the overall quality of the protein provided by the quail breast meat: in general, HI2 meat showed a slightly better amino acid profile than HI1, especially when considering arginine and threonine (*p* < 0.05) of the essential amino acids and glycine, glutamic acid, and serine (*p* < 0.05) of the nonessential amino acids.

The inclusion of fish offal in the conventional growing substrate for HI larvae influenced the main FA classes (Table 1), but differently from the hypothesis, the saturated fatty acid (SFA) proportion increased (72.0% vs. 68.9% SFAs for HI2 and HI1 larvae, respectively), whereas total polyunsaturated fatty acids (PUFAs) decreased (6.99% vs. 12.6% PUFAs for HI2 and HI1 larvae, respectively). Such a reduction involved only the *n*-6 PUFA fraction, whereas the *n*-3 one showed a three-fold increase (1.57% vs. 0.53% *n*-3 PUFAs for HI2 and HI1 larvae, respectively), thus resulting in a nutritionally valuable improvement in the *n*-6/*n*-3 ratio (3.45 vs. 22.9 for HI2 and HI1 larvae, respectively).

The FA profile of the quail breasts (% of total FAMEs) showed remarkable differences depending on the presence and type of HI included in the experimental diets (Table 5). The inclusion of HI larvae increased the total SFA proportion compared to the C diet, with HI2 breasts showing a greater SFA proportion even compared to HI1 breasts (32.0% vs. 40.3% vs. 42.8% for C, HI1, and HI2 breast meat, respectively; *p* < 0.001). The higher SFA content of quail breasts derived from HI-fed quails compared to C was attributable to the increasing percentages of C12:0, C14:0, and C16:0 (*p* < 0.001) FAs (Table 1). In addition, the monounsaturated FA (MUFA) proportion increased in the breasts of HI-fed quails, with the differences attributable to the different growing substrates used to farm *H. illucens* larvae (18.8% vs. 22.7% and 22.2% for C, HI1, and HI2 breasts, respectively; *p* < 0.001). The MUFAs affected by the dietary treatments to the greatest extent were the C14:1, C15:1, and C16:1 (*p* < 0.01) FAs, although these specific FAs were present at low concentrations. As a result of the dietary treatment (Table 1), the overall PUFA percentage differed in the C, HI1, and HI2 groups (Table 5). Specifically, C showed the highest level compared to HI1 and HI2 breasts, which did not differ from each other (44.1% vs. 32.0% and 30.4% for C, HI1, and HI2 breast meat, respectively; *p* < 0.001). Despite this, the two HI breasts showed different PUFA compositions: HI1 breasts highlighted a consistent decrease in both the *n*-6 as well as the *n*-3 PUFA fractions compared to the C group (*p* < 0.001), whereas HI2 breasts exhibited the most intense lowering of the *n*-6 PUFAs but a consistent increase of the *n*-3 PUFA fraction, which was similar to that of the C group. The loss of *n*-6 and the concomitant increase in the *n*-3 PUFAs consistently reduced the *n*-6/*n*-3 ratio, which was the lowest in the HI2 breasts and the highest in the HI1 breasts (11.2 vs. 14.0 vs. 6.56 *n*-6/*n*-3 for C, HI1, and HI2 breasts, respectively; *p* < 0.001).

As a result of the changes in the FA profile of quail breasts belonging to the different dietary treatments, the health indexes of the quail breasts were also influenced (Table 5): the atherogenicity index (AI) and the thrombogenicity index (TI) increased (*p* < 0.001), thus worsening with the dietary inclusion of HI (both types). Furthermore, according to the different growing substrates used to farm larvae, different magnitudes in these changes were observed: HI1 breasts showed a lower AI but a higher TI compared to HI2 breasts. As a result of a growing SFA proportion in HI breasts compared to C, the hypocholesterolemic/hypercholesterolemic ratio (HH ratio) decreased, thus worsening, and the peroxidability index (PI) decreased, too, thus making the meat of HI1 and HI2 quails less susceptible to oxidative phenomena than that of the C group (61.7 and 66.5 vs. 81.9 for HI1, HI2, and C breast meat, respectively; *p* < 0.001). 

Overall, the dietary inclusion of HI larvae reared on different growing substrates into quails’ diets did not negatively impact the sensory profile of the breast meat, which showed similar traits in the three groups (Table 6). The only exception was with regard to some specific textural attributes: juiciness and fibrousness. Specifically, the HI2 meat had the lowest juiciness compared to C and HI1 (4.53 vs. 5.01 and 4.90 for HI2, C, and HI1 breast meat, respectively; *p* < 0.001). As a consequence, HI2 meat also exhibited the highest fibrousness, followed by HI1 and C meat (5.28 vs. 5.05 vs. 4.79 for HI2, HI1, and C breast meat, respectively; *p* < 0.001). As indicated in Table 7, the off-odors and off-flavors were not affected by the experimental diets.

The results of the retail display trial (Table 8) showed that the dietary treatment did not play a role in affecting the broiler quail breast meat traits: Drip loss percentage, overall colorimetric characteristics, and oxidative status showed similar results throughout the retail display trial. The only exception was the lightness (L*) value measured at day 0 of the retail display, when HI2 meat had a higher value compared to C, with HI1 being intermediate (51.6 vs. 50.6 vs. 49.0 for HI2, HI1, and C breast meat, respectively; *p* < 0.05).

## 4. Discussion

The physical breast meat traits observed in the present research (Table 2) were overall satisfactory and in line with values reported for quails [27]. The highest cooking loss shown by HI2 meat was similar to that observed by Cullere et al. [8]. In the latter, breast meat derived from quails fed with the highest dietary inclusion level (15%) of a partly defatted *H. illucens* larvae meal showed the highest cooking loss. In this case, a lower meat pH was also observed and it was hypothesized as causative agent of the higher cooking loss: as the pH value approaches the isoelectric point of proteins, their water holding capacity reduces thus generating a higher moisture loss. However, HI2 meat in the present trial had the same pH of the other experimental groups, and thus a causative agent for the observed different cooking loss percentage must be searched for elsewhere. 

Even if the experimental diets HI1 and Control of the present experiment had slightly different absolute protein contents (23.9 and 24.3 g/100 g feed for HI1 and Control diets, respectively) [18], the lower protein content observed in the breast meat of the HI1 quails compared to the Control group could not be directly attributable to this factor. It is now well established that not all nitrogen present in insects originates from protein: insects’ exoskeleton contain chitin, a polysaccharide containing N atoms, which typically falls within the amount of nitrogen quantified by the common Kjeldahl method. On the other hand, during the larvae’s sclerotization phase of development, different proteins harden the cuticle (exoskeleton) by linking the chitin fibers. This particular structural arrangement of the insect cuticle makes such proteins indigestible to animals [28]. Up to a couple of years ago, these factors were ignored in the formulations of animal diets that included insects, thus technically resulting in diets that were only apparently isonitrogenous because the protein content of insects was generally overestimated [29]. To solve this drawback, further research on this topic resulted in a recent recommendation of using a specific nitrogen conversion factor for insects (5.62) [30]. This above-mentioned issue could be the main reason for the observed difference in the protein content of HI1 and Control quail breasts. Interestingly, for HI2 meat, the same finding was not applicable. It is known that different rearing substrates can determine diverse development rates in *H. illucens* larvae [31]: the HI2 larvae could have been slightly less developed than the HI1 larvae, and this could have led to different sclerotization stages of the cuticle in the two HI larvae. As a consequence, the amount of indigestible protein could have been different in the two HI larvae, thus leading to the observed results in breast meat protein content.

The results regarding the amino acid content of breast meat (Table 4) further emphasized the above-mentioned speculations: HI1 meat had an absolute lower amino acid content compared to the Control meat (19.3 compared to 21.6 g/100 g meat), which determined the lower protein percentage in the HI1 group compared to the Control meat. However, in a previous study by Cullere et al. [9], the same finding was not observed, as quails receiving increasing dietary inclusion levels (up to 15%) of a partly defatted *H. illucens* larvae meal had similar protein and thus amino acid contents. This apparent discrepancy could be explained by the fact that, in Cullere et al. [9], the tested insect source was a defatted meal derived from the same aged larvae that had been fed the same diets. Previous literature has shown that the defatting process concentrates protein and amino acids, therefore determining a higher amount of amino acids compared to full-fat larvae [32]. When defatted *H. illucens* larvae were incorporated into different avian species such as quail [9], chicken [33], and Barbary partridge [10], the protein quantity of the derived meat was similar in the experimental groups. 

The inclusion of dietary ingredients with cholesterol up to a certain threshold in poultry diets should only moderately affect meat because liver lipid metabolism can decrease cholesterol de novo synthesis as well as increase the transformation and transportation rate as a response to the dietary level: this mechanism is essential to guarantee optimal animal health, welfare, and growth [34]. Despite this, independent of the growing substrate, the inclusion of 10% HI larvae into quail diets increased the cholesterol content of the breasts compared to the Control group. Literature studies considering this aspect are still limited, and available results are controversial: In a work by Cullere et al. [35] testing *H. illucens* fat as a soybean oil replacer in finisher broiler chickens, a 100% substitution produced breast meat with the same cholesterol content as the Control group. A similar finding was observed by Cullere et al. [9] in broiler quails fed either a 10% or 15% *H. illucens* meal. In the latter, however, a numerical but not significant increase in meat cholesterol contents going from the lowest to the highest inclusion level (71.6, 73.3, and 74.9 mg/100 g meat at 0%, 10%, and 15% inclusion levels, respectively) was noted. In past experiments studying quail metabolism, it emerged that these birds respond particularly quickly and intensely to dietary cholesterol content and that they can develop atherosclerosis relatively fast, with relevant differences depending on sex and genetics [36,37]. Based on such speculations, it would be interesting to investigate the effects of genetics, sex, and type of insect product fed to quails on their cholesterol metabolism.

The fact that HI1 meat showed the highest meat lipid content was unexpected, especially considering that the dietary fat content was similar in the three experimental groups and almost identical in the HI1 and HI2 diets [18]. A possible reason to explain this could come from the results of the digestibility trial [18], where the HI1 quails showed the highest apparent digestibility of starch and metabolizable energy, which could explain the higher meat lipid content observed in the present research. However, this finding was not confirmed by previous studies on quail [9], chicken [33,38], or Barbary partridge [10] fed with different inclusion levels of *H. illucens* larvae protein meal or fat. 

The results of the present research demonstrate once more that the FA composition of the *H. illucens* larvae can only be moderately affected by the farming substrate. In this case, exploiting fish offal waste to modulate the larvae growing media improved the healthiness of their FA profile. In fact, the *n*-3 proportion increased (+34% compared to HI1 larvae), almost exclusively due to eicosapentaenoic (EPA) and docosahexaenoic (DHA) acids, which are FAs typically found in marine fish sources that have important implications in human health mainly due to their positive effects on cardiometabolic risk factors [39]. Despite this, the overall FA profile was similar in HI1 and HI2 larvae and coherent with the typical FA profile that has been reported in the literature for this insect species: rich in lauric (C12:0), myristic (C14:0), and palmitic (C16:0) FAs for the SFA fraction and with oleic (C18:1 *n*-9) and linoleic (C18:2 *n*-6) FAs as the main MUFAs and PUFAs, respectively [10,35]. The successful increase in the *n*-3 proportions in the *n*-3 HI-fed larvae was in agreement with previous studies testing the inclusion of fish offal [17], fishmeal [40], or brown algae (*Ascophyllum nodosum*) in a growing substrate [41]. The high SFA content of *H. illucens* larvae is linked to their peculiar adult stage: In fact, after pupation, the buccal apparatus regresses, and then adults cannot feed anymore. For this reason, to survive, they rely on energy reserves accumulated during their larval stage. Furthermore, it seems that dietary PUFAs are primarily used by the larvae as an energy source (oxidation) and that extra energy is stored in their bodies mainly as SFAs and MUFAs [40], thus explaining why their FA profile was poorly represented by PUFAs. As a direct effect of larvae FA profiles, when the 10% insect meal was included into the quails’ diets, their FA profiles were characterized by a 28% SFA increase and an overall reduction in MUFA (−5.5%) and PUFA (−57%) proportions. This had a direct effect on the FA composition of the meat obtained from quails fed the experimental diets. In fact, SFAs increased by 30% and PUFAs decreased by 29%. Even if dietary SFA intake in humans should be moderate, as they are associated with increased cardiovascular disease risk [42], it must be stressed that SFAs play important roles in normal cellular and tissue metabolism and, among other things, they are essential constituents of cell membrane phospholipids, structurally part of important cell signaling molecules, and are involved in gene expression and in the modulation of cholesterol, fatty acid, and triacylglycerol synthesis and lipoprotein assembly, secretion, and clearance [43]. Specifically, lauric acid, which accounts for the greatest part (about 65%) of larvae’s SFA percentage and for about 13% of the meat SFA proportion, is converted in human and animal bodies to monolaurin, which in turn aids in preventing viral, bacterial, and protozoal infections [44]. Furthermore, 6–12 medium-chain FAs in general are efficiently absorbed, digested, and beta-oxidized in the gut and are thus very efficient energy substrates for both livestock and humans [45]: they are reported to improve the gut’s health thanks to their intrinsic antibacterial and antiviral properties [46]. The addition of HI to the quails’ diets reduced the overall dietary MUFA content, but the observed trend in the meat was not coherent in this sense: The meat of the HI1 and HI2 groups had about +15% MUFAs compared to the Control group. This outcome agreed with previous observations on quails fed increasing levels of a partly defatted *H. illucens* larvae meal [9] but not with those reported on broiler chickens whose dietary content of soybean oil was replaced by 50% and 100% *H. illucens* fat [35]. This could highlight, on the one hand, a particular intrinsic capability of quails to desaturate and elongate SFAs into MUFAs. On the other hand, it further corroborates the hypothesis that diets characterized by low fat and high carbohydrates have a role in the upregulation of the activity of the lipogenic enzyme ∆9 desaturase [47]. In fact, the Control diet of the present study slightly differed in terms of fat and starch contents compared to those including the 10% HI larvae [18]. Regarding meat PUFAs, it was observed that modulating HI larvae substrate with fish offal was a successful way to improve the *n*-3 PUFA proportion in the meat of HI-fed quails and thus lower the breasts’ *n*-6/*n*-3 ratio, which is considered a sort of target index for a meat to be considered as healthy [48]. Specifically, as a result of the HI substrate modulation, in the present experiment a reduction of ~53% and ~41% of the *n*-6/*n*-3 ratio in HI2 quails compared to the HI1 and Control quails, respectively, was noted.

Research dealing with the effect of meat obtained from insect-fed poultry on its sensory characteristics is still limited. Despite the fact that *H. illucens* larvae meals are characterized by a peculiar flavor [49], existing knowledge seems to indicate that the impact of this emerging feed ingredient on the sensory traits of animal products is limited [11]. The sensory profile of quail meat and that of eggs obtained from quails fed with *H. illucens* larvae meal (up to a 15% inclusion level) did not differ from the Control group without insect meal [9,15]. In addition, the use of *H. illucens* fat as an alternative fat source for broiler chickens did not affect the sensory traits of the derived meat compared to chicken fed with a conventional soybean oil diet [35]. In this context, the present research is the first highlighting significant changes in the textural traits of quail meat: As meat of the HI2 treatment displayed the highest cooking loss, it was not surprising that meat juiciness and fibrousness were the lowest and the highest, respectively. This is because, regardless of the considered animal species, cooking loss has a great influence on the juiciness of meat [50]. On the other hand, it was unexpected that higher fibrousness would be indicated for the HI1 meat compared to the Control. Furthermore, HI1 meat was also the richest in lipids, which should have had a positive effect on meat sensory characteristics, including textural and sensory perception [51]. 

The results of the quail breasts subjected to the retail display trial are in agreement with Schiavone et al. [38], who evaluated the impact of feeding *H. illucens* fat to poultry on their meat quality, which was evaluated during refrigerated storage. Meat physical traits are responsible for its visual appearance, which has a key impact on consumer choice at purchase [52]. In view of this, the fact that the meat of the three dietary treatments showed similar drip loss and comparable colorimetric characteristics is of utmost importance for marketing purposes. The highest L* value at day 0 of refrigerated storage observed in the HI2 meat was not expected, since the same result was not noted when studying the physical breast meat characteristics. In past studies, it was found that *H. illucens* larvae contain carotenoids (about 2.00–2.15 mg/kg), which can positively affect the yolk yellowness of eggs produced by quails and hens fed with this larvae meal [15,53]. No mention of a possible effect on meat color/lightness, however, was found in literature that considered poultry species fed different inclusion levels and products derived from *H. illucens* larvae. Interestingly, despite the intense modifications in the FA profile of the quail meat, its oxidative status remained unaffected throughout the retail display trial, ensuring that the meat, in this sense, was completely satisfactory from a qualitative point of view.

## 5. Conclusions

The present experiment showed that it is possible to exploit a valuable nutrient source that is generally considered a waste to partly improve the fatty acid profile of *H. illucens* larvae: In fact, the use of fish offal to farm *H. illucens* larvae provided a substantial enrichment of the HI with *n*-3 fatty acids. This, in turn, led to a lower *n*-6/*n*-3 ratio in broiler quail meat, which was the primary objective of the present work. The main remaining drawback, however, is still the SFA proportion (that is, the preponderant larvae lipid fraction), which directly reflects on the FA profile of quail meat. Therefore, if the ultimate goal is to produce meat with a healthy lipid profile, it would be interesting to test the direct inclusion of a PUFA-rich feed ingredient (i.e., linseed) in quail diets in combination with an *n*-3 enriched HI meal. Based on the present findings, further research to investigate the role of this insect source in the poultry metabolism of fats and cholesterol is also warranted.

## Figures and Tables

**Table 1:** Fatty acid profile (% of total FAMEs) of the dried *Hermetia illucens* (HI) larvae reared on layer mash (HI1) or on 50:50 layer mash and fish offal (HI2) and of the experimental diets: Control and Control diet including either 10% HI1 or HI2.

Fatty Acids	Dried Larvae		Experimental Diets		
	HI1	HI2	Control	HI1	HI2
C10:0	0.95	1.02	0.00	0.62	0.68
C12:0	44.2	48.3	0.06	29.4	33.1
C14:0	7.98	6.88	0.09	5.15	4.66
C15:0	0.09	0.03	0.00	0.08	0.13
C16:0	13.6	13.4	11.4	12.8	12.9
C17:0	0.09	0.11	0.10	0.14	0.11
C18:0	1.74	2.06	2.64	1.80	1.97
C20:0	0.23	0.04	0.07	0.07	0.06
C22:0	0.02	0.02	0.14	0.07	0.06
C23:0	0.00	0.02	0.05	0.04	0.05
C24:0	0.00	0.12	0.06	0.00	0.17
Total SFAs	68.9	72.0	14.6	50.1	53.8
C14:1	0.16	0.20	0.00	0.11	0.13
C15:1	0.05	0.02	0.00	0.00	0.00
C16:1	2.39	3.82	0.10	1.60	2.73
C17:1	0.13	0.16	0.11	0.09	0.17
C18:1 *n*-9	13.7	13.3	24.3	17.3	16.9
C18:1 *n*-11	1.11	1.05	1.39	0.71	0.87
C20:1 *n*-9	0.11	0.09	0.39	0.24	0.21
C22:1 *n*-9	0.08	0.03	0.34	0.11	0.10
C24:1 *n*-9	0.00	0.00	0.47	0.19	0.12
Total MUFAs	17.7	18.7	27.1	20.4	21.2
C18:2 *n*-6	11.0	4.28	51.3	25.1	18.8
C18:3 *n*-6	0.02	0.05	0.07	0.04	0.04
C18:3 *n*-3	0.44	0.19	4.37	1.16	1.35
C20:2 *n*-6	0.08	0.63	0.05	0.06	0.04
C20:3 *n*-6	0.02	0.04	0.05	0.05	0.04
C20:3 *n*-3	0.02	0.07	0.07	0.06	0.06
C20:4 *n*-6	0.05	0.12	0.06	0.06	0.09
C20:5 *n*-3	0.07	1.00	0.08	0.05	0.58
C22:2 *n*-6	0.96	0.29	0.40	0.64	0.22
C22:6 *n*-3	0.00	0.32	0.00	0.00	0.10
Total PUFAs	12.6	6.99	56.4	27.3	21.3
UFAs/SFAs	0.44	0.36	5.68	0.95	0.79
*n*-6	12.1	5.42	51.9	26.0	19.2
*n*-3	0.53	1.57	4.51	1.26	2.09
*n*-6/*n*-3	22.9	3.45	11.5	20.6	9.20
Identified FAs (%)	99.7	97.9	98.2	97.8	96.4

FAME = fatty acid methyl ester; MUFA = monounsaturated fatty acid; PUFA = polyunsaturated fatty acid; SFA = saturated fatty acid; UFA = unsaturated fatty acid; FA = fatty acid.

**Table 2:** Effect of the dietary inclusion of *Hermetia illucens* dried larvae farmed on different growth substrates (HI1, HI2) on the physical traits of broiler quails’ breast meat.

Physical Traits	Experimental Groups ^1^			*p*-Value	RSD ^2^
	C	HI1	HI2		
No.	37	39	39		
pH	5.62	5.59	5.63	0.2346	0.14
L*	49.6	49.9	50.1	0.6205	3.02
a*	2.44	2.28	2.11	0.2374	1.21
b*	9.32	9.48	9.42	0.8382	1.66
Thawing loss, %	11.3	10.9	10.9	0.9173	3.25
Cooking loss, %	20.3 ^b^	20.0 ^b^	22.0 ^a^	0.0380	3.36
Total loss, %	31.6	30.9	32.9	0.2393	4.65
WBSF, kg/cm^2^	11.3	10.9	10.4	0.0680	1.72

^1^ HI1: control diet incorporated with 10% dried *Hermetia illucens* larvae reared on a substrate made of 100% layer mash; HI2: control diet incorporated with 10% dried *Hermetia illucens* larvae reared on a substrate made of 50% layer mash and 50% fish offal; ^2^ RSD: residual standard deviation; L*: lightness; a*: redness; b*: yellowness; WBSF: Warner–Bratzler shear force; ^a,b^ values within a row with different superscripts differ significantly at *p* < 0.05.

**Table 3:** Effect of the dietary inclusion of *Hermetia illucens* dried larvae farmed on different growth substrates (HI1, HI2) on the proximate composition (%), cholesterol, and heme iron contents (mg/100 g meat) of broiler quails’ breast meat.

Chemical Traits	Experimental Groups ^1^			*p*-Value	RSD ^2^
	C	HI1	HI2		
No.	12	12	12		
Water	74.5	74.5	74.6	0.4220	0.25
Protein	23.9 ^a^	23.5 ^b^	23.7 ^ab^	0.0143	0.26
Lipids	4.89 ^B^	5.27 ^A^	4.93 ^B^	0.0003	0.23
Ash	2.31	2.29	2.29	0.9777	0.26
Cholesterol	68.7 ^Bb^	74.1 ^A^	73.6 ^ABa^	0.0031	3.96
Heme iron ^3^	0.64	0.60	0.66	0.3044	0.87

^1^ HI1 = diet supplemented with 10% dried *Hermetia illucens* larvae reared on layer mash; HI2 = diet supplemented with 10% dried *Hermetia illucens* larvae reared on 50:50 layer mash and fish offal; ^2^ RSD = residual standard deviation; ^3^ heme iron analysis was conducted on No. = 10 fresh breast meat samples/treatment; ^a,b^ means in a row with different superscripts differ significantly (*p* < 0.05); ^A,B^ means in a row with different superscripts differ significantly (*p* < 0.01).

**Table 4:** Effect of the dietary inclusion of *Hermetia illucens* dried larvae farmed on different growth substrates (HI1, HI2) on the amino acid content (g/100 g meat) of broiler quails’ breast meat.

Amino Acid Content	Experimental Diets			Experimental Groups ^1^			*p*-Value	RSD ^2^
	C	HI1	HI2	C	HI1	HI2		
No.				12	12	12		
% Protein	92.6	80.6	87.0	89.5 ^a^	81.8 ^b^	83.4 ^ab^	0.0226	6.62
Essential amino acids								
Arginine	1.52	1.21	1.49	1.46 ^a^	1.31 ^b^	1.33 ^ab^	0.0238	0.14
Histidine	0.52	0.45	0.52	0.85 ^A^	0.74 ^B^	0.74 ^B^	0.0003	0.07
Isoleucine	0.87	0.89	0.90	0.96	0.90	0.95	0.0710	0.07
Leucine	1.85	1.75	1.77	2.01 ^Aa^	1.76 ^B^	1.83 ^ABb^	0.0017	0.16
Lysine	0.61	0.66	0.82	0.88	0.87	1.00	0.1862	0.18
Methionine	0.25	0.23	0.19	0.54 ^A^	0.47 ^B^	0.45 ^B^	0.0008	0.05
Phenylalanine	1.58	1.32	1.28	1.81 ^A^	1.22 ^B^	1.25 ^B^	0.0006	0.38
Threonine	0.80	0.68	0.78	0.95 ^a^	0.86 ^b^	0.87 ^ab^	0.0429	0.09
Valine	0.87	0.83	0.89	0.98	0.91	0.97	0.0609	0.07
Nonessential amino acids								
Alanine	1.12	1.07	1.12	1.32	1.24	1.28	0.2052	0.12
Aspartic acid	2.49	1.85	2.14	2.10	1.88	1.94	0.0582	0.22
Glycine	1.25	1.10	1.15	1.23 ^a^	1.12 ^b^	1.12 ^ab^	0.0175	0.11
Glutamic acid	4.10	3.08	3.60	3.16 ^a^	2.86 ^b^	2.91 ^ab^	0.0357	0.30
Proline	1.54	1.44	1.37	0.93	0.90	0.94	0.5473	0.08
Hydroxyproline	0.42	0.33	0.34	0.54 ^a^	0.48 ^ab^	0.46 ^b^	0.0287	0.07
Serine	1.25	1.00	1.16	0.95 ^a^	0.86 ^b^	0.86 ^ab^	0.0250	0.09
Tyrosine	1.01	0.97	1.04	0.96	0.89	0.89	0.2037	0.11

^1^ HI1 = diet supplemented with 10% dried *Hermetia illucens* larvae reared on layer mash; ^2^ HI2 = diet supplemented with 10% dried *Hermetia illucens* larvae reared on 50:50 layer mash and fish offal; ^3^ RSD = residual standard deviation; ^a,b^ means in a row with different superscripts differ significantly (*p* < 0.05); ^A,B^ means in a row with different superscripts differ significantly (*p* < 0.01).

**Table 5:** Effect of the dietary inclusion of *Hermetia illucens* dried larvae farmed on different growth substrates (HI1, HI2) on the fatty acid profile (% of total FAMEs) of broiler quail breast meat.

Fatty Acids	Experimental Groups ^1^			*p*-Value	RSD ^2^
	C	HI1	HI2		
No.	12	12	12		
C10:0	0.01 ^C^	0.11 ^B^	0.14 ^A^	<0.0001	0.03
C12:0	0.22 ^C^	4.58 ^B^	6.49 ^A^	<0.0001	1.07
C14:0	0.31 ^B^	2.48 ^A^	2.79 ^A^	<0.0001	0.37
C16:0	16.6 ^B^	19.2 ^A^	19.5 ^A^	<0.0001	0.93
C17:0	0.06	0.04	0.04	0.5978	0.06
C18:0	13.1^a^	12.3 ^ab^	12.0 ^b^	0.0109	0.88
C20:0	0.11	0.08	0.10	0.5825	0.06
C22:0	0.13	0.11	0.13	0.1049	0.03
C23:0	0.57 ^B^	0.74 ^A^	0.37 ^C^	<0.0001	0.13
C24:0	0.87 ^B^	0.60 ^A^	1.12 ^C^	<0.0001	0.83
Total SFAs	32.0 ^C^	40.3 ^B^	42.8 ^A^	<0.0001	1.86
C14:1	0.00 ^B^	0.31 ^A^	0.30 ^A^	<0.0001	0.05
C15:1	0.06 ^B^	0.08 ^AB^	0.12 ^A^	0.0012	0.03
C16:1	1.02 ^B^	3.16 ^A^	3.09 ^A^	<0.0001	0.45
C17:1	0.20	0.17	0.19	0.2548	0.04
C18:1 *n*-9	15.4 ^b^	17.0 ^a^	16.3 ^ab^	0.0136	1.30
C18:1 *n*-11	1.71 ^A^	1.50 ^B^	1.80 ^A^	<0.0001	0.29
C20:1 *n*-9	0.34	0.30	0.31	0.8064	0.13
C22:1 *n*-9	0.12	0.12	0.12	0.9615	0.05
Total MUFAs	18.8 ^B^	22.7 ^A^	22.2 ^A^	<0.0001	1.64
C18:2 *n*-6	32.0 ^A^	22.1 ^B^	20.4 ^C^	<0.0001	1.21
C18:3 *n*-6	0.12	0.10	0.13	0.4937	0.05
C18:3 *n*-3	1.46 ^A^	0.48 ^B^	0.54 ^B^	<0.0001	0.13
C20:2 *n*-6	0.17	0.20	0.23	0.3251	0.09
C20:3 *n*-6	0.42	0.46	0.40	0.1811	0.08
C20:3 *n*-3	0.36 ^B^	0.61 ^A^	0.56 ^A^	<0.0001	0.12
C20:4 *n*-6	7.50 ^A^	6.74 ^A^	5.03 ^B^	<0.0001	0.99
C20:5 *n*-3	0.26 ^B^	0.21 ^B^	0.99 ^A^	<0.0001	0.13
C22:2 *n*-6	0.21	0.21	0.16	0.5621	0.13
C22:6 *n*-3	1.59 ^Ab^	0.89 ^B^	1.97 ^Aa^	<0.0001	0.34
Total PUFAs	44.1 ^A^	32.0^B^	30.4 ^B^	<0.0001	2.24
*n*-6	40.5 ^A^	29.8 ^B^	26.4 ^C^	<0.0001	1.88
*n*-3	3.66 ^A^	2.19 ^B^	4.06 ^A^	<0.0001	0.50
*n*-6/*n*-3	11.2 ^B^	14.0 ^A^	6.56 ^C^	<0.0001	1.63
PI	81.9 ^A^	61.7 ^B^	66.5 ^B^	<0.0001	7.51
AI	0.29 ^C^	0.62 ^B^	0.71 ^A^	<0.0001	0.07
TI	0.74 ^B^	1.04 ^Aa^	0.94 ^Ab^	<0.0001	0.09
HH ratio	3.46 ^A^	2.20 ^B^	2.03 ^B^	<0.0001	0.23
Identified FAs, %	95.0	95.0	95.4		

^1^ HI1 = diet supplemented with 10% dried *Hermetia illucens* larvae reared on layer mash; ^2^ HI2 = diet supplemented with 10% dried *Hermetia illucens* larvae reared on 50:50 layer mash and fish offal; ^3^ RSD = residual standard deviation; PI = peroxidability index; AI = atherogenicity index; TI = thrombogenicity index; HH ratio = hypocholesterolemic/hypercholesterolemic ratio; ^a,b^ means in a row with different superscripts differ significantly (*p* < 0.05); ^A,B^ means in a row with different superscripts differ significantly (*p* < 0.01).

**Table 6:** Effect of the dietary inclusion of *Hermetia illucens* dried larvae farmed on different growth substrates (HI1, HI2) on the sensory scores of broiler quails’ breast meat.

Descriptors	Experimental Groups ^1^			*p*-Value	RSD ^2^
	C	HI1	HI2		
No.	16	16	16		
Odor intensity	5.10	5.31	5.48	0.1446	0.52
Off-odor intensity	0.90	1.15	1.03	0.1748	0.37
Flavor intensity	5.13	5.52	5.29	0.1232	0.52
Off-flavor intensity	1.15	1.14	1.11	0.9725	0.46
Juiciness	5.01 ^A^	4.90 ^A^	4.53 ^B^	0.0008	0.33
Toughness	4.76	4.53	4.55	0.2638	0.44
Chewiness	6.24	6.22	6.41	0.1471	0.28
Fibrousness	4.79 ^Bb^	5.08 ^ABa^	5.28 ^A^	0.0006	0.31

Sensory attributes were scored on numerical and continuous scales from 0 (the lowest score for each attribute) to 10 (the highest score for each attribute); No. = 6 breasts of the Control (C) group were used for the training sessions; ^1^ HI1 = diet supplemented with 10% dried *Hermetia illucens* larvae reared on layer mash; ^2^ HI2 = diet supplemented with 10% dried *Hermetia illucens* larvae reared on 50:50 layer mash and fish offal; ^3^ RSD = residual standard deviation; ^a,b^ means in a row with different superscripts differ significantly (*p* < 0.05); ^A,B^ means in a row with different superscripts differ significantly (*p* < 0.01).

**Table 7:** Effect of the dietary inclusion of *Hermetia illucens* dried larvae farmed on different growth substrates (HI1, HI2) on the off-odors and off-flavors (% of total tested samples) of broiler quails’ breast meat.

Descriptors	Experimental Groups ^1^			*p*-Value	RSD ^2^
	C	HI1	HI2		
No.	16	16	16		
Off-odors					
Game meat	18.8	6.25	6.25	0.5926	1.79
Metallic	6.25	0.00	0.00	1.0000	2.04
Liver	31.3	50.0	31.3	0.5930	1.60
Oil/fat	6.25	25.0	31.3	0.2788	3.28
Peanut/hazelnut	12.5	0.00	6.25	0.7650	2.13
Rancid	0.00	0.00	12.5	0.3110	4.17
Off-flavors					
Game meat	6.25	18.8	18.8	0.6810	1.34
Metallic	18.8	0.00	0.00	0.1000	6.40
Liver	37.5	75.0	56.3	0.1152	4.57
Oil/fat	6.25	25.0	6.25	0.3342	3.43
Peanut/hazelnut	12.5	12.5	6.25	1.0000	0.45
Rancid	0.00	0.00	0.00	-	-

No. = 6 breasts of the C group were used for the training sessions. ^1^ HI1 = diet supplemented with 10% dried *Hermetia illucens* larvae reared on layer mash; ^2^ HI2 = diet supplemented with 10% dried *Hermetia illucens* larvae reared on 50:50 layer mash and fish offal.

**Table 8:** Effect of the dietary inclusion of *Hermetia illucens* dried larvae farmed on different growth substrates (HI1, HI2) on a seven-day retail display trial of broiler quail breast meat.

Retail Display Traits	Experimental Groups ^1^			*p*-Value	RSD ^2^
	C	HI1	HI2		
No.	12	12	12		
Drip loss, %	6.05	4.83	6.67	0.0525	1.63
Day 0					
pH	5.60	5.54	5.54	0.4075	0.10
L*	49.0 ^b^	50.6 ^ab^	51.6 ^a^	0.0357	2.15
a*	2.07	2.83	3.02	0.2025	1.17
b*	10.3	10.6	11.5	0.2161	1.54
TBARs, mg MDA/kg meat	1.52	1.51	1.53	0.2179	0.02
Day 8					
pH	5.90	5.90	6.01	0.1424	0.14
L*	47.7	46.4	46.7	0.6531	3.27
a*	2.68	4.06	3.46	0.1207	1.44
b*	10.4	10.7	11.7	0.4268	2.26
TBARs, mg MDA/kg meat	1.84	1.75	1.87	0.6852	0.30

^1^ HI1 = diet supplemented with 10% dried *Hermetia illucens* larvae reared on layer mash; ^2^ HI2 = diet supplemented with 10% dried *Hermetia illucens* larvae reared on 50:50 layer mash and fish offal; ^3^ RSD = residual standard deviation; ^a,b^ means in a row with different superscripts differ significantly (*p* < 0.05); TBARs = thiobarbituric acid-reactive substances; MDA = malondialdehyde.
